# PIP-PACA: An Interpretable Image Classification Framework via Prototype-Aware Clustering Attention

**DOI:** 10.3390/jimaging12070308

**Published:** 2026-07-08

**Authors:** Xinyuan Jia, Yanling Li, Yihui Wang

**Affiliations:** School of Mathematics and Statistics, Qinghai Minzu University, Xining 810007, China; jxywyyx369@163.com (X.J.); 19513583380@163.com (Y.W.)

**Keywords:** interpretability, image classification, prototype learning, attention mechanism

## Abstract

Image classification interpretability remains a fundamental challenge in the field of computer vision. Despite the remarkable improvements achieved by deep neural networks in classification accuracy, their decision-making processes are often opaque, which limits their applicability in high-stakes scenarios requiring reliability and transparency. Prototype-based methods, such as PIP-Net, address this issue by establishing explicit correspondences between input images and semantic prototypes, thereby enabling an intuitive, evidence-based reasoning paradigm. However, these approaches still suffer from insufficient global context modeling and underutilization of structural relationships among prototypes. To address these limitations, this paper proposes an interpretable image classification model termed PIP-PACA, which is built upon a prototype-aware clustering attention mechanism. In contrast to conventional Transformer architectures based on self-attention, the proposed PACA module introduces a set of learnable cluster centers to project feature representations into a prototype space. Global information is then captured via a bidirectional attention mechanism between features and prototypes. This design is inherently aligned with the principles of prototype learning while reducing the computational complexity from quadratic to linear. Furthermore, a normalization operation is incorporated during the feature extraction stage to enhance the stability of feature distributions and improve the reliability of prototype matching. Extensive experimental results demonstrate that the proposed method not only preserves the interpretability of the original framework but also achieves notable improvements in classification accuracy, sparsity, and prototype purity. These findings validate the effectiveness and superiority of the clustering-based attention mechanism within the prototype learning paradigm.

## 1. Introduction

Deep neural networks have achieved remarkable success in image classification; however, their decision-making processes remain largely opaque. Although modern architectures such as convolutional neural networks (CNNs) and Vision Transformers can attain high predictive accuracy, they typically operate as black-box models, limiting their applicability in safety-critical and high-risk domains where transparency and trust are essential. Therefore, interpretability at both the model level and the individual prediction level is of great importance.

Recent research in interpretable image classification has explored prototype-based models, which explain predictions by measuring the similarity between input regions and learned semantic prototypes. Among these approaches, PIP-Net introduces an intuitive reasoning paradigm aligned with human cognition, where predictions are formed by aggregating evidence from sparse and semantically meaningful visual parts. Nevertheless, existing prototype-based methods still suffer from limited global context modeling and insufficient structural organization among prototypes, which may lead to unstable predictions and suboptimal semantic consistency.

In contrast to post hoc explanation methods, this work treats interpretability as an intrinsic property of the model. According to the theory of recognition-by-components [1], human visual perception recognizes objects by decomposing them into meaningful parts. Meanwhile, Occam’s razor [2] suggests that simpler explanations with fewer assumptions should be preferred when multiple hypotheses are equally valid. Furthermore, Dempster–Shafer (D–S) evidence theory [3] indicates that excessive evidence may reduce the confidence of the final decision rather than improve it. From an information-theoretic perspective, larger subsets introduce higher uncertainty. These principles collectively imply that a small number of clear and informative evidence components is beneficial for accurate and reliable classification.

Motivated by these observations, we propose an image classification framework that emphasizes interpretability, sparsity, and semantic alignment. The proposed method requires only image-level labels and does not rely on additional part annotations, while still learning meaningful and discriminative visual parts. These parts correspond to interpretable image patches that are consistent with human visual perception [4]. To enhance sparsity and global context modeling, normalization layers and attention modules are incorporated before and after the feature extraction stage of PIP-Net, enabling the model to capture more informative features through contextual reasoning. As a result, the model achieves efficient classification by activating fewer discriminative components [5]. PIP-Net provides inherent global interpretability through a sparse scoring mechanism, where prototypes serve as positive evidence for each class. This design enables a transparent decision-making process, as predictions are supported by accumulated evidence rather than suppressed by negative contributions. Such a mechanism is consistent with human visual reasoning, where conclusions are drawn based on supporting evidence. Moreover, inspired by hierarchical processing in biological vision systems, the proposed model decomposes inference into multiple stages and integrates both local and global features to provide comprehensive evidence for classification.

Interpretable prototype-based models, such as ProtoTree [6], ProtoPNet [7], ProtoPool [8], and ProtoPShare [9], suffer from the so-called semantic gap [10,11]. Specifically, in part-based prototype models (e.g., ProtoPNet and ProtoTree), this gap refers to the inconsistency between similarity in the latent space and similarity in the input space. A primary cause of this issue is that these models regularize interpretability only at the class level. They implicitly assume that all samples within the same class share identical prototypes, thereby overlooking intra-class variability.

**Figure 1:**
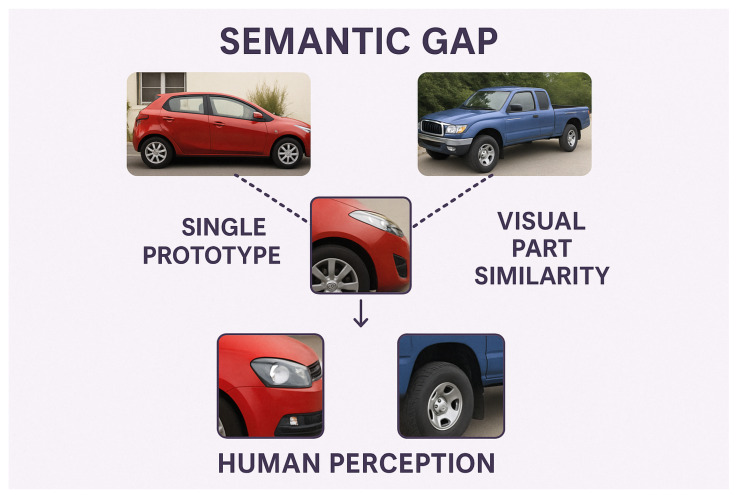
Human visual perception recognizes two cars, yet in prototype learning only one car prototype can be learned.

The limitation of existing approaches lies in their inability to adequately capture the diversity of visual parts or features within a single class. To illustrate this issue, consider a binary classification task (as shown in Figure 1), where the objective is to distinguish between images containing “a red car or a blue truck” and those that do not. In this scenario, an ideal model should learn separate prototypes for red cars and blue trucks. However, existing models may instead learn a single prototype to represent both concepts, particularly when the model is constrained to use a small number of prototypes. Such behavior leads to a mismatch between the model’s perception of visual similarity and human visual cognition. Humans naturally perceive red cars and blue trucks as distinct concepts, whereas the model may incorrectly treat them as a single prototype. This discrepancy not only reduces the intuitiveness of the model’s explanations, making its decision-making process difficult to interpret in human-understandable terms, but also degrades overall interpretability, as the learned prototypes fail to align with meaningful visual concepts.

PIP-Net effectively mitigates the discrepancy between the latent space and the pixel space, while maintaining strong interpretability aligned with human visual perception. In PIP-Net, learned prototype-based parts are connected to class predictions through a sparse linear layer, enabling direct interpretation of the relationship between prototypes and classes.

The weights of the linear layer are constrained to be non-negative, ensuring that the presence of class-relevant prototypes provides only positive evidence for the corresponding class. The class score is computed as the weighted sum of activated prototypes, forming a transparent and additive scoring mechanism. For local interpretability, the model identifies the spatial locations where prototypes are activated within the input image. For global interpretability, the model exposes sparse associations between classes and their corresponding prototypes in the decision layer.

This explicitly interpretable linear formulation establishes a direct correspondence between prototypes and classification outcomes, thereby avoiding the unreliable explanations commonly observed in post hoc or purely local interpretability methods. However, PIP-Net primarily relies on local visual evidence and lacks the ability to effectively model global contextual information. Consequently, the model exhibits limited sensitivity to the overall prototype distribution and broader image context, such as background information.

To address the limitation of PIP-Net in insufficiently incorporating global image information during prediction, we propose an improved model that integrates a clustering-based attention mechanism. Unlike standard self-attention, the proposed Prototype-Aware Clustering Attention (PACA) module introduces a set of learnable cluster centers to regulate feature interactions in a structured and semantically meaningful manner. This design enables effective global information aggregation while preserving the interpretability of prototype-based reasoning. Although PIP-Net performs well in local prototype discrimination, its prediction process primarily relies on local evidence—classification is triggered by the presence of prototypes, with limited consideration of prototype quantity and background context. As a result, while accuracy improves, semantic consistency among prototypes remains suboptimal. By incorporating the PACA mechanism, the proposed model is able to capture local features while simultaneously integrating global contextual information. Experimental results show that this combination not only improves classification accuracy but also significantly enhances prediction consistency. These findings validate the effectiveness of clustering-based attention in strengthening global perception while retaining the advantages of PIP-Net in local prototype reasoning.

Extensive experiments on fine-grained benchmarks demonstrate that the proposed method not only improves classification accuracy but also achieves superior performance in prototype sparsity, semantic consistency, and predictive reliability. These results highlight clustering-driven attention as a principled approach for enhancing the interpretability of prototype-based models.

### Our Contributions Are Summarized as Follows:

1. We propose a novel interpretable image classification framework, termed PIP-PACA, which unifies prototype learning with a clustering-based attention mechanism. Unlike existing approaches that directly incorporate Transformer modules, the proposed method introduces an attention mechanism that is structurally consistent with prototype-based reasoning.
2. We introduce a lightweight Prototype-Aware Clustering Attention (PACA) module, which replaces dense self-attention with clustering-mediated interactions. This design enables efficient global context modeling while remaining consistent with prototype-based interpretability, and reduces the computational complexity from $O\left(N^{2}\right)$ to $O\left(N\right)$.
3. We introduce a normalization-enhanced feature processing strategy that improves the consistency of feature distributions without disrupting the semantic structure of prototypes, leading to faster convergence and improved training stability.
4. Extensive experiments demonstrate that the proposed method outperforms conventional prototype-based models in terms of classification accuracy, prototype sparsity, and prototype purity, while maintaining strong interpretability.

## 2. Related Work

Meike Nauta et al. [6] proposed the Neural Prototype Tree (ProtoTree), an inherently interpretable deep learning method for fine-grained image recognition. It combines prototype learning with decision trees to form a globally interpretable model. Each internal node of ProtoTree contains a trainable prototype, and the routing of an input image is determined by the presence or absence of this prototype. The leaf nodes subsequently learn class distributions. Through techniques such as pruning and binarization, ProtoTree achieves strong interpretability while maintaining high accuracy, providing both global and local explanations with significantly fewer prototypes than conventional prototype-based networks. Chaofan Chen et al. [7] introduced the Prototypical Part Network (ProtoPNet), which mimics human reasoning by decomposing images into representative parts and aggregating their evidence for classification. The model identifies discriminative image patches and performs classification based on their combined evidence, requiring only image-level labels without part-level annotations. Experimental results show that ProtoPNet achieves accuracy comparable to non-interpretable counterparts. Moreover, when multiple ProtoPNets are combined into a larger ensemble, the performance becomes competitive with state-of-the-art deep models while retaining superior interpretability. Jiaqi Wang et al. [12] proposed TesNet, which inserts a class-specific transparent embedding space at the top of a CNN, spanned by orthogonal bases on a Grassmann manifold. This design directly maps high-order image patches to traceable semantic concepts, where each basis vector corresponds to a specific image patch and forms a scorecard-style reasoning mechanism. An Orth+Sep regularization is introduced to enforce intra-class orthogonality and inter-class separation, achieving semantic disentanglement. On the CUB-200 and Stanford Cars datasets, TesNet improves accuracy by 1–2% over ProtoPNet, with an additional 1–2% gain when combined with multiple networks, while maintaining full interpretability. Mengqi Xue et al. [13] proposed ProtoPFormer to address the issue that Vision Transformer (ViT)-based prototypes are easily distracted by background information. The method introduces a global masking mechanism and local Gaussian constraints to encourage prototypes to focus on foreground object parts. Experiments on CUB, Dogs, and Cars datasets show that ProtoPFormer consistently outperforms baseline methods such as ProtoPNet by 1–4% with only 6–9 M parameters, while clearly localizing key regions such as bird heads and car wheels. Wang et al. [14] proposed MCPNet, an inherently interpretable model that learns meaningful concept prototypes across multiple feature map levels using Centered Kernel Alignment (CKA) loss and an energy-based weighted PCA mechanism. Unlike existing methods that rely on high-level semantics from the last feature map, MCPNet provides comprehensive multi-level explanations while maintaining classification accuracy, and demonstrates improved generalization in few-shot scenarios. Dawid Rymarczyk et al. [8] proposed ProtoPool, an interpretable prototype-based model with three key innovations for positive reasoning: sharing prototypes across classes to significantly reduce their number; enabling automatic and fully differentiable prototype-to-class assignment; and introducing a novel focal similarity function that emphasizes salient visual features. ProtoPool achieves state-of-the-art accuracy on the CUB-200-2011 and Stanford Cars datasets, substantially reduces the number of prototypes, and is shown—through theoretical analysis and user studies—to capture more salient features than competing methods. Dawid Rymarczyk et al. [9] further proposed ProtoPShare, an extension of ProtoPNet that allows prototype sharing across classes. By applying data-dependent similarity-based pruning, ProtoPShare significantly reduces the number of required prototypes while maintaining baseline accuracy and reveals inter-class prototype similarities. Compared to ProtoPNet trained with shared prototypes alone, ProtoPShare achieves higher accuracy through a two-stage training process (initial training followed by prototype pruning) and a data-driven merging and pruning strategy.

Meike Nauta et al. [15] introduced the Patch-based Intuitive Prototype Network (PIP-Net), an interpretable image classification model based on self-supervised learning, where learned prototype parts exhibit stronger alignment with human visual perception. PIP-Net connects prototypes to class predictions via a sparse linear layer that can be interpreted as a scorecard. The model relies only on image-level labels, requires no part annotations, can handle out-of-distribution samples, and is capable of rejecting uncertain inputs by indicating “unknown” predictions. Its self-supervised pretraining employs contrastive learning with alignment and consistency losses [16], enabling prototypes to capture meaningful semantics and reducing the semantic gap between latent and pixel spaces. Haibo Jin et al. [17] proposed PIPNet (Pixel-in-Pixel Net) for facial landmark detection, addressing issues such as redundant computation in heatmap regression, limited coordinate regression accuracy, and poor cross-domain generalization. The model performs Pixel-in-Pixel (PIP) regression to simultaneously predict confidence scores and offsets on low-resolution feature maps, avoiding repeated upsampling and enabling real-time inference on CPUs (35.7 FPS). It further introduces a Neighbor Regression Module (NRM) to enhance local geometric consistency and proposes a self-training curriculum (STC) to iteratively refine pseudo-labels using unlabeled cross-domain data, representing an early exploration of generalized semi-supervised learning (GSSL) in landmark detection. Shreyasi Pathak et al. [18] proposed PEF-Coh, a framework for systematically evaluating the interpretability of prototype-based models in mammography for breast cancer diagnosis. The framework quantifies prototype quality across seven dimensions, including relevance, purity, uniqueness, coverage, and localization accuracy, evaluating models such as ProtoPNet, BRAIxProtoPNet++, and PIP-Net. The study reveals that 28–36% of prototypes are unrelated to regions of interest (ROI), with low purity and insufficient class coverage, while PIP-Net demonstrates superior compactness and fine-grained purity compared to other methods. Meike Nauta et al. [19] further applied PIP-Net to medical image classification tasks, including fracture detection and skin cancer diagnosis, validating its interpretability and calibration capability on real clinical data. By visualizing prototype parts (e.g., fracture regions and artifacts), the study shows that PIP-Net can reveal dataset shortcuts, such as color calibration charts in the ISIC dataset being misinterpreted as benign features. Notably, it demonstrates for the first time that human intervention can directly correct model bias by disabling artifact-related prototypes, improving the specificity for malignant images from 9% to 65.7% without retraining the entire model. Zhu et al. [20] presented a non-parametric part prototype learning framework for interpretable image classification. By clustering deep features extracted from foundation vision models, their method learns semantically distinctive object parts in a non-parametric fashion, yielding more diverse and comprehensive explanations compared to conventional ProtoPNets. They further introduced Distinctiveness Score and Comprehensiveness Score to quantitatively evaluate explanation quality.

The aforementioned methods all aim to enhance the interpretability and intuitiveness of image classification models. Through various prototype-based approaches, they provide decision-making processes that are more transparent and better aligned with human understanding. While ProtoTree, ProtoPNet, ProtoPool, ProtoPShare, and PIP-Net each introduce innovations in prototype learning and utilization, they also exhibit inherent limitations. Specifically, the first five methods lack a clear semantic correspondence between learned prototypes and human cognition. This so-called semantic gap—referring to the inconsistency between similarity in the latent space and the input space—has also been identified by other studies in prototype-based learning. Although PIP-Net partially addresses this issue, it still suffers from several limitations: (1) the model can only detect the presence of prototypes and is unable to account for the frequency of prototype occurrences as a discriminative feature; (2) its classification performance on non-fine-grained datasets (e.g., PartImageNet [21]) remains slightly inferior to that of conventional black-box models; and (3) although the number of activated prototypes has been reduced, it is still not sufficiently compact.

Motivated by these limitations, we aim to design a model that can capture global feature representations while incorporating a feature filtering mechanism during inference. The Transformer [22], a deep learning architecture based on the self-attention mechanism, has been widely adopted in natural language processing (NLP). It processes input and output sequences through an encoder–decoder architecture and leverages multi-head self-attention to model contextual dependencies. Owing to its strong representation capability, Transformer-based models have recently been extended to computer vision (CV) tasks. Empirical studies demonstrate that Transformers achieve performance comparable to or superior to other architectures, such as convolutional neural networks (CNNs) and recurrent neural networks (RNNs), across various visual benchmarks. Due to their high performance and reduced reliance on visual inductive biases, Transformers have attracted increasing attention in the computer vision community.

In recent years, Transformer-based models in the vision domain have been primarily represented by Vision Transformer (ViT) and Swin Transformer. ViT [23,24,25], proposed by Google, is a pure Transformer-based architecture for visual tasks. It divides an image into a sequence of fixed-size patches, which are then mapped into a high-dimensional embedding space via a linear projection layer and fed into the Transformer. When pretrained on large-scale datasets such as JFT-300M, ViT achieves performance comparable to or exceeding state-of-the-art convolutional neural networks (CNNs) on multiple image classification benchmarks. However, ViT suffers from high computational complexity when processing high-resolution images and exhibits weaker generalization on smaller datasets compared to conventional CNNs. Swin Transformer [26,27,28], proposed by Microsoft Research, addresses these limitations by introducing a hierarchical architecture along with a shifted window mechanism. By restricting self-attention computation to local windows while enabling cross-window connections, Swin Transformer achieves linear computational complexity and can efficiently handle high-resolution images. It demonstrates strong performance across various vision tasks, including image classification, object detection, and semantic segmentation, significantly outperforming prior state-of-the-art methods. Overall, ViT and Swin Transformer represent two important directions for applying Transformer architectures to computer vision. While ViT establishes the feasibility of pure Transformer models for image classification, Swin Transformer improves upon its limitations in computational efficiency and scalability, further enhancing performance and applicability in vision tasks.

More recently, hybrid architectures that integrate convolutional neural networks (CNNs) with Transformers have demonstrated significant performance improvements in visual tasks. Several studies have validated the effectiveness of this direction. CoAtNet [29] (Dai et al., 2021) systematically combines convolution and self-attention across different stages, effectively balancing local receptive fields and global context modeling. CvT [30] (Wu et al., 2021) further refines the ViT architecture by incorporating convolutional operations into both token embedding and Transformer blocks, enhancing fine-grained feature representation while improving training stability and convergence speed. Building upon this, Next-ViT [31] (Li et al., 2022) introduces the Next Hybrid Strategy (NHS), which alternates between efficient Next Convolution Blocks (NCB) and Next Transformer Blocks (NTB) at each stage, enabling effective modeling of both local details and global dependencies. This design significantly improves accuracy while reducing inference latency across tasks such as image classification, object detection, and semantic segmentation.

Subsequently, PaCa-ViT [32] addresses the quadratic complexity and interpretability challenges of Vision Transformers by proposing an end-to-end Patch-to-Cluster Attention (PaCa) mechanism. Unlike conventional patch-to-patch attention, this approach maps image patches into a predefined cluster space, achieving linear computational complexity. Moreover, the cluster assignment matrix provides inherent interpretability. Experimental results show that PaCa-ViT not only significantly improves inference efficiency on high-resolution tasks (e.g., semantic segmentation) but also outperforms strong baselines such as Swin Transformer and PVT on benchmarks like ImageNet. Collectively, these studies demonstrate that integrating CNNs with Transformers can effectively mitigate their respective limitations while leveraging their complementary strengths, offering a promising direction for developing high-performance, interpretable, and efficient vision models.

Given the strong performance of PIP-Net and the need to control model complexity, we adopt a lightweight Prototype-Aware Clustering Attention (PACA) block and integrate it with PIP-Net for feature fusion.

While PaCa-ViT also employs clustering to reduce attention complexity, three key differences distinguish our PACA module. First, PaCa-ViT operates in a pure Transformer architecture with token-mixing, whereas PACA preserves the grid structure of convolutional features and avoids tokenization, maintaining spatial locality critical for prototype matching. Second, PaCa-ViT uses clustering as a computational shortcut for self-attention, while PACA is designed specifically for prototype learning: the cluster centers serve as semantic anchors that directly align with the prototype space, making the mechanism inherently interpretable rather than merely efficient. Third, PaCa-ViT’s cluster assignment is a byproduct of attention computation, whereas PACA’s assignment matrix explicitly groups semantically similar regions, directly supporting the evidence-based reasoning paradigm of prototype models.

## 3. Model Architecture

Building upon the PIP-Net framework, we introduce a Prototype-Aware Clustering Attention (PACA) mechanism to develop a novel interpretable image classification model. The proposed model preserves the original prototype-based reasoning structure while incorporating a clustering-based global modeling module at the feature representation stage. The overall architecture consists of feature extraction, normalization, clustering-based attention modeling, and prototype matching. Given an input image *I*, feature maps are first extracted using a convolutional neural network backbone: $X=f_{\mathrm{backbone}}\left(I\right)$, $X\in R^{C\times H\times W}$, where *C* denotes the number of channels, and H,W represent spatial dimensions. The extracted feature map preserves local structural information and serves as the basis for subsequent prototype matching.

Feature responses across different channels often exhibit varying magnitudes, which may affect the stability of similarity computation. In prototype learning and clustering-based modeling, fluctuations in similarity distributions can lead to inconsistent feature assignments, causing semantically similar regions to be mapped to different clusters across samples. To mitigate this issue, Group Normalization (GN) [33] is applied before clustering: $X^{\left(1\right)}=GN\left(X\right)$.

Group Normalization partitions channels into groups and performs normalization within each group. Unlike batch normalization, it does not depend on batch statistics and remains stable under small-batch training. This operation aligns feature distributions across channels, leading to more stable similarity computation and more reliable clustering assignments.

The normalized feature is then fed into the PACA module. Instead of adopting pairwise self-attention as in Transformers, PACA introduces a set of learnable cluster centers as intermediate representations. Feature interactions are performed indirectly through these cluster centers, avoiding direct pairwise computations across spatial positions.

The feature map is first reshaped into a sequence: $X_{f}\in R^{N\times C},N=H\times W$. A set of K learnable cluster centers is defined as: $C=\left\{c_{1},c_{2},\ldots ,c_{K}\right\},c_{k}\in R^{C}$. The similarity between features and cluster centers is computed as: $s_{ik}=x_{i}^{⊤}c_{k}$. The assignment weights are obtained via a softmax function:

(1) $$a_{ik}=\frac{\exp(s_{ik}/T)}{\sum_{j=1}^{K}\exp\left(s_{ij}/T\right)}$$

where *T* is a temperature parameter. The assignment matrix encodes the association between features and cluster centers, where semantically similar regions tend to be grouped into the same cluster. Feature aggregation is then performed in the cluster space: $f_{k}=\sum_{i=1}^{N}a_{ik}\cdot x_{i}$. The aggregated cluster features capture global semantic information. These features are subsequently projected back to the original feature space: ${\hat{x}}_{i}=\sum_{k=1}^{K}a_{ik}\cdot f_{k}$.

This process establishes long-range dependencies across spatial locations while preserving the original spatial structure. The reconstructed features are fused with the input features through a residual connection:

(2) $$\tilde{X}=X^{\left(1\right)}+\alpha \cdot \hat{X}$$

Since clustering operations may alter the feature distribution, an additional Group Normalization is applied to stabilize the feature scale: $Z=\mathrm{GN}(\tilde{X})$.

The two normalization steps jointly ensure that features maintain consistent scale both before and after clustering-based interaction, preventing numerical instability such as feature explosion or collapse and leading to more stable training dynamics.

With the enhanced feature representation *Z*, the model proceeds to the prototype matching stage. Each class is represented by a set of semantic prototypes. The similarity between feature maps and prototypes is computed. For the *j*-th prototype, the activation is defined as: $p_{j}=\max_{i}\mathrm{si}m\left(z_{i},p_{j}\right)$. All prototype activations form a prototype presence vector: $p=[p_{1},p_{2},\ldots ,p_{M}]$.

Instead of using a standard linear classifier, we introduce a constrained nonlinear transformation [34]. For class *c*, the output is defined as:

(3) $$o_{c}=\log\left(\sum_{j=1}^{M}(p_{j}w_{j,c}{)}^{2}+1\right)$$

where $w_{j,c}\geq 0$ denotes the contribution weight of the *j*-th prototype to class *c*. The computation process first applies weighting to the prototype responses, then performs a squaring operation, and finally compresses the numerical range via a logarithmic function.

This formulation amplifies the contribution of highly activated prototypes while suppressing weak responses. As a result, classification decisions are dominated by a small number of key prototypes, leading to more concentrated and interpretable evidence.

Compared to linear classification, this nonlinear mapping improves discriminative capability while preserving interpretability. Each class prediction can still be decomposed into a combination of prototype-based evidence, with reweighted contributions emphasizing more informative prototypes.

**Figure 2:**
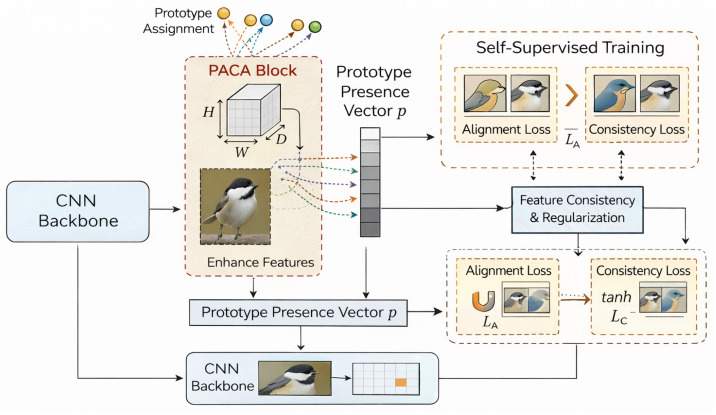
Overall architecture of the PIP-PACA model. The figure illustrates the PACA module, the prototype presence vector, and the alignment loss and classification loss. The overall objective additionally includes the tanh loss (see Equation (5)), which ensures each prototype is activated at least once per mini-batch, though not explicitly shown in the figure.

As illustrated in Figure 2, the PACA module does not alter the prototype reasoning mechanism but introduces structured global information modeling at the feature level. Feature interactions are mediated through cluster centers, avoiding the quadratic complexity of pairwise attention. The computational complexity is reduced from $O\left(N^{2}\right)$ to $O\left(NK\right)$. When K≪N, the computational overhead is significantly diminished, while the feature interaction mechanism remains consistent with prototype learning.

## 4. Prototype Self-Supervised and Supervised Training

PIP-Net employs a self-supervised learning strategy [35,36] to learn semantically meaningful prototypes without requiring part-level annotations. During the pretraining stage, the classification layer is frozen, and the model focuses on optimizing prototypes to capture semantic similarity. This ensures that visually distinct prototypes remain well separated in the latent space. Additionally, positive pairs are generated via data augmentation, incorporating human perceptual consistency into training.

PIP-Net employs a contrastive loss to align the latent representations of two augmented views of the same image patch. The similarity between the two representations is measured by the dot product and maximized. The alignment loss is defined as:

(4) $$L_{A}=-\frac{1}{HW}\sum_{(h,w)\in H\times W}\log\left(z_{h,w,:}^{\prime }\cdot z_{h,w,:}^{\prime \prime }\right)$$

PIP-Net employs a contrastive loss to align the latent representations of two augmented views of the same image patch. Specifically, given an input image, two augmented versions are generated through random transformations (e.g., color jittering, random cropping, and horizontal flipping). These two views, denoted as $z_{h,w,:}^{\prime }$ and $z_{h,w,:}^{\prime \prime }$, are fed through the network independently, and the alignment loss encourages their latent representations to be similar at corresponding spatial positions. PIP-Net further introduces a tanh loss function to ensure that each prototype appears at least once in a mini-batch, thereby encouraging the model to utilize all prototypes. The loss is defined as:

(5) $$L_{T}\left(p\right)=-\frac{1}{D}\sum_{d}^{D}\log\left(\tanh\left(\sum_{b}^{B}p_{b}\right)+\epsilon \right)$$

To process the features, a softmax normalization [37] is applied to normalize the correlations between each spatial position and all prototypes into a probability distribution: $z_{h,w,:}=\mathrm{Soft}\max\left(z_{h,w,:}\right)$. Then, a max-pooling operation is applied to obtain the strongest response of each prototype in the image:

(6) $$p_{d}=\max_{h,w}z_{h,w,d}$$

In this way, a vector *p* is obtained to represent the presence degree of all prototypes in the image. After completing prototype pretraining, the final linear layer is unfrozen, and the entire model is jointly optimized. A classification loss term $L_{C}=-\sum_{c=1}^{C}y_{c}\log\left({\hat{y}}_{c}\right)$ is introduced to improve classification performance. This loss corresponds to the standard negative log-likelihood between the predicted output $\hat{y}$ and the one-hot encoded ground-truth label *y*. The loss primarily updates the weights of the linear layer, while also fine-tuning the prototypes to better align with downstream classification tasks. The overall objective of the second training stage in PIP-Net is defined as: $\lambda_{C}L_{C}+\lambda_{A}L_{A}+\lambda_{T}L_{T}$.

## 5. PACA Module Mechanism

The effectiveness of the proposed method lies in its structural integration, where an efficient global modeling mechanism is embedded into a framework that inherently provides a clear interpretability pathway. Traditional self-attention emphasizes exhaustive interactions across all spatial positions. However, such fully connected interactions do not explicitly account for semantic relevance, which may lead to spurious associations between target regions and complex backgrounds, resulting in dispersed attention distributions.

**Figure 3:**
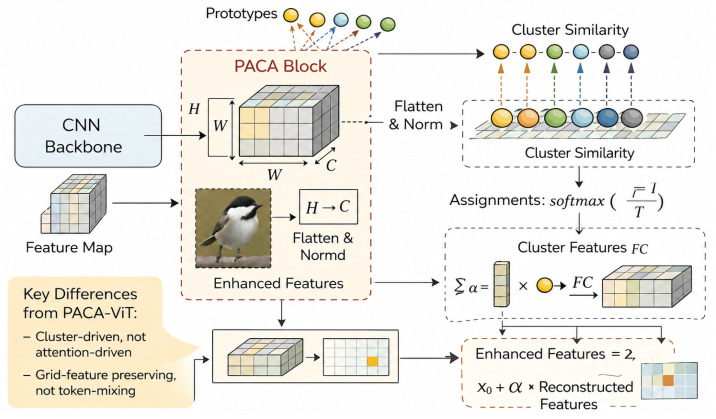
Internal structure of the PACA module. The figure illustrates the clustering-driven feature enhancement pipeline, including feature flattening and normalization, cluster similarity computation, soft assignment, and feature reconstruction. The overall computation additionally incorporates a residual connection to stabilize gradient propagation and preserve the original grid structure, which is indicated in simplified form in the figure.

In contrast, the PACA module organizes feature interactions through a clustering-based mechanism, where information propagation is centered around a set of cluster centers. These centers act as filters during computation, grouping semantically similar regions into the same clusters while naturally separating irrelevant regions. Compared to conventional attention mechanisms, the proposed clustering attention can be viewed as a variant of attention, whose core idea is to organize features into semantic clusters via clustering. Therefore, the module can be characterized as a clustering-driven feature enhancement structure. An illustration of the clustering attention mechanism is shown in Figure 3.

Taking bird images as an example, regions such as the head, wings, and tail are aggregated into a small number of relevant clusters, while background regions (e.g., forest) are assigned to separate clusters. The spatial structure of features is preserved, but their representations become more concentrated in the semantic space. As a result, interference from background noise is reduced, and representations of semantically similar regions become more consistent.

After this process, the features fed into the prototype matching layer are more discriminative, with key regions more prominently highlighted. The learned prototypes are cleaner, and class separation becomes more stable. The overall computation still follows the original prototype activation and weighting mechanism of PIP-Net, without introducing additional non-interpretable components. The final predictions remain traceable through prototype responses and class-specific weights, ensuring a transparent decision-making process.

## 6. Computational Complexity Analysis

A key advantage of the PACA module lies in its computational efficiency, which stems from its interaction paradigm between spatial positions and cluster centers. To illustrate this property, we compare it with the standard self-attention mechanism. Assume that the input feature map contains *N* spatial positions, where N=H×W, with *C* channels and batch size *B*. Standard self-attention requires computing pairwise correlations among all spatial positions, resulting in an attention matrix of size N×N. The core computation arises from matrix multiplication between queries and keys, resulting in a complexity of $O\left(B\cdot N^{2}\cdot C\right)$. As the input resolution increases, the number of spatial positions grows proportionally to the image area. If *N* increases by a factor of 4, the total computation becomes 16 times larger. Thus, the computational cost scales quadratically with *N*, which is the primary reason for the high overhead of self-attention in high-resolution scenarios.

In contrast, the PACA module adopts a different attention mechanism. Instead of performing pairwise comparisons between spatial positions, it computes the similarity between *N* spatial positions and *M* cluster centers. The computational cost of this step is $O\left(B\cdot N\cdot M\cdot C\right)$. Subsequently, the aggregated cluster features are projected back to the *N* spatial positions according to the assignment relationships, which also incurs a complexity of $O\left(B\cdot N\cdot M\cdot C\right)$. Since *M* is a predefined hyperparameter and typically much smaller than *N*, it can be treated as a constant in analysis. Therefore, the overall complexity of the PACA attention mechanism can be approximated as $O\left(B\cdot N\cdot C\right)$, which scales linearly with the number of spatial positions.

This difference becomes more pronounced under high-resolution inputs. The computational cost of self-attention increases quadratically with *N*, resulting in rapid growth, whereas the cost of PACA grows linearly with *N*, leading to a more moderate increase. Theoretically, the ratio of their computational complexity is approximately $\frac{M}{N}$. When N⩾M, the advantage of the PACA module becomes increasingly significant. This complexity characteristic makes the module more efficient in practical deployment and more suitable for integration with convolutional backbone networks.

## 7. Scoring Mechanism Design and Implementation

In PIP-Net, the inference pipeline proceeds as follows. Features extracted from the backbone are first normalized to obtain $X^{\left(1\right)}$. The normalized features are then fed into the PACA block, where clustering-based attention enables global context modeling and high-level semantic feature extraction. The resulting representations are fused through a residual connection to produce the enhanced feature *Z*. The feature *Z* is subsequently compared with prototypes to obtain the prototype presence vector *p*, which is combined with the classification weights *W* to produce class scores. This design maintains a direct correspondence between feature representations and classification decisions, preserving interpretability throughout the inference process.

During training, to improve model performance and enforce sparsity regularization [38,39], a specialized normalization strategy is adopted. Specifically, the class score is computed as: $o_{c}=\log\left(\sum_{j=1}^{M}(p_{j}w_{j,c}{)}^{2}+1\right)$. This logarithmic normalization offers several advantages. First, the compression of large values reduces the model’s reliance on excessively high prototype responses, thereby mitigating overconfidence. Second, the relative amplification and penalization of small values encourage the model to suppress weights that are less relevant to the classification task, promoting sparsity.

This design not only improves generalization performance but also ensures that the resulting explanations are more compact and interpretable. By effectively optimizing the weights and reducing the influence of redundant features during training, the model produces more accurate and interpretable predictions at inference time.

## 8. Experiments and Results

We evaluate our model on standard benchmark datasets in the prototype learning domain, including CUB-200-2011 [40] (200 fine-grained bird species) and Stanford Cars [41] (196 car categories).

Unlike conventional image classification studies that primarily focus on maximizing predictive accuracy, this work treats interpretability as a first-class objective. Accordingly, in addition to standard classification metrics, we evaluate our model from multiple interpretability perspectives, including prototype sparsity and prototype purity.

We mainly compare our method with existing prototype-based models, as they share the same goal of providing intrinsic interpretability. While black-box models such as CNNs and Vision Transformers may achieve higher accuracy, they do not offer transparent reasoning processes and are therefore not the primary focus of this study.

### 8.1. Experimental Setup

The experimental environment consists of both hardware and software configurations. The hardware setup is based on the Ubuntu 22.04 operating system and is equipped with an NVIDIA GeForce RTX 4090 GPU (24 GB) and an Intel Xeon Platinum 8352V processor (2.10 GHz). The software environment is built on Python 3.12 and utilizes the PyTorch 2.7.0 framework for model implementation and training. CUDA 12.8 is employed to enable GPU-accelerated computation. This configuration provides sufficient computational resources for efficient model training and evaluation.

### 8.2. Experimental Settings

In our experiments, ConvNeXt-Tiny [42] is adopted as the backbone architecture, and evaluations are conducted on the CUB and Cars datasets.

The number of cluster centers is set to M=280, and the temperature coefficient is set to T=0.6. Specifically, the value of *M* is determined based on the observation that PIP-Net retains approximately 320 non-zero prototypes on the CUB-200-2011 dataset. Selecting a cluster center count slightly below this value facilitates effective aggregation of features toward semantic prototypes, while avoiding the loss of discriminative information caused by excessive clustering. The temperature coefficient T=0.6 is determined via grid search over the range {0.1,0.3,0.6,1.0}. This value yields a moderately smooth soft-assignment distribution, balancing training stability in the early stages with clear cluster boundaries after convergence. These two parameters determine the representation capacity and assignment behavior of the clustering-based attention mechanism. Specifically, a larger number of cluster centers enables finer-grained semantic grouping, while a smaller temperature value leads to more concentrated feature assignments.

The training procedure follows the two-stage strategy of PIP-Net. In the first stage, prototype pretraining is performed for 10 epochs. During this stage, the classification layer remains frozen, and the model focuses on learning stable prototype representations. In the second stage, end-to-end joint training is conducted for 100 epochs. In this stage, all network parameters are optimized jointly, allowing feature extraction, clustering-based attention, and prototype-based classification to be collaboratively refined. Images are resized to 224×224 pixels and augmented using the TrivialAugment method [43] to improve model generalization. During training, prototype pretraining is first conducted for 10 epochs, followed by full training of the entire model for 100 epochs. The model is optimized using the Adam optimizer with a cosine annealing learning rate schedule. The loss weights are set as $\lambda_{C}=\lambda_{T}=2$ and $\lambda_{A}=5$. Our model is built upon the PIP-Net framework. To ensure fair and direct comparability with the baseline, the loss weight parameters are inherited directly from the original PIP-Net paper $\lambda_{C}=\lambda_{T}=2,\lambda_{A}=5$. This configuration has been extensively validated by PIP-Net on the CUB-200-2011 and Stanford Cars datasets, and is empirically shown to effectively balance classification accuracy, prototype alignment, and prototype utilization. Re-tuning these weights might yield local improvements for our model, but would compromise the fairness of cross-model comparisons, making it impossible to attribute performance gains solely to the proposed PACA module. We therefore retain the original settings.

### 8.3. Performance Evaluation

In the experiments, the proposed method builds upon PIP-Net by introducing Group Normalization and the PACA block, enabling improved classification accuracy while maintaining high sparsity and compact local explanations. To provide a broader perspective on interpretable image classification, we additionally compare with MCPNet, a recent prototype-based framework based on multi-level concept representations. Unlike PIP-Net and PIP-PACA, whose prototypes are sparsely and independently activated as part detectors, MCPNet employs Multi-Level Concept Prototypes (MCPs) that model normalized probability distributions over concepts at each semantic level. Consequently, the two methods follow fundamentally different prototype paradigms, making metrics such as prototype count, explanation granularity, and sparsity inherently incomparable. The results are summarized in Table 1. It is worth noting that ProtoPNet employs a fully connected classification layer and therefore does not exhibit sparsity in the same sense.

As shown in Table 1, on the CUB-200-2011 dataset, the proposed prototype-based model with clustering attention (PIP-PACA) achieves an accuracy of 83.2%, representing a 4% improvement over the original PIP-Net. Compared with the recent MCPNet, PIP-PACA further improves the classification accuracy by 5.0 percentage points. This gain is significant given the already strong baseline performance. The global explanation size is slightly reduced from 316 to 312. Moreover, the total number of prototypes is reduced from 768 to 312, corresponding to a 59.4% reduction.

On the Cars dataset, PIP-PACA achieves an accuracy of 89.1%, improving upon the baseline by 6.3%. It also outperforms MCPNet by 7.5 percentage points in terms of Top-1 accuracy. A similar compression effect is observed in the global explanation size, while the total number of prototypes is reduced from 768 to 252, corresponding to a 67.2% reduction. These results indicate that the proposed model enhances the effectiveness of prototype representations. In addition, sparsity is slightly improved. Here, sparsity is defined as the proportion of zero-valued connections in the linear classification layer. A higher sparsity implies that the model relies on fewer prototypes for decision-making, resulting in more concise and focused explanations.

Compared to the original PIP-Net, the proposed model improves accuracy without increasing the number of prototypes, achieving simultaneous gains in both global explanation compactness and Top-1 accuracy. More importantly, the reduction in prototype count and the increase in sparsity reflect the effectiveness of the PACA module in semantic aggregation. The improved model also retains the interpretability advantages of the original framework. For instance, local explanations remain compact, requiring only a small number of prototypes to understand the model’s predictions. This compactness facilitates efficient validation and enhances user trust. Furthermore, the model maintains the ability to handle out-of-distribution samples, preserving the open-set recognition capability [44] of PIP-Net and providing a more reliable solution for practical applications.

As shown in Table 1, ProtoPNet exhibits substantial performance degradation across all key metrics relative to PIP-Net. Given that the present work aims to investigate the potential for improvement upon the PIP-Net framework rather than conduct an exhaustive comparison with all prototype-based approaches, subsequent analyses of prototype semantic quality—including purity assessment and consistency visualization—are restricted to PIP-Net and PIP-PACA. This deliberate scope ensures both methodological comparability and a targeted evaluation of the proposed architectural contributions.

### 8.4. Ablation Study: Synergistic Effects of Structural Components

**Figure 4:**
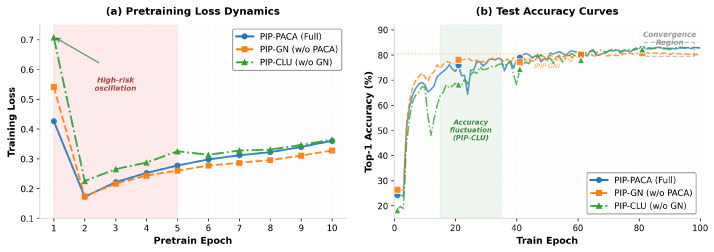
Comparison of training dynamics across ablation configurations. Left (**a**): Pretraining loss curves, with the shaded region (epochs 1–5) indicating the high-risk oscillation period, where PIP-CLU (without GN) exhibits notably higher initial loss than other configurations. Right (**b**): Test accuracy curves, with the green shaded region (epochs 15–40) marking the accuracy fluctuation interval of PIP-CLU, and the dashed box denoting the convergence region (epochs 80–100). The three configurations are: PIP-PACA (full model with PACA module and dual GN), PIP-GN (PACA removed, dual GN retained), and PIP-CLU (GN removed, PACA retained).

To validate the individual contributions and synergistic effects of the PACA module and the normalization strategy, a systematic ablation study is conducted, with results summarized in Table 2. As illustrated in Figure 4a, during the pretraining stage (epochs 1–10), the configuration without Group Normalization (PIP-CLU) exhibits pronounced loss oscillation: its initial loss reaches 0.71, which is 65% higher than that of the full model (PIP-PACA) at 0.43, and the loss curve presents two distinct inflection points within the first five epochs. This observation indicates that although the PACA module possesses global semantic aggregation capability, the soft assignment of cluster centers produces unreliable attention weights due to scale drift in the absence of stable feature distributions.

From the perspective of final convergence performance, the three configurations demonstrate an interesting separation pattern. After removing the PACA module (PIP-GN), the model degenerates into a baseline form based on local prototype matching, with its Top-1 accuracy decreasing by 2.4 percentage points (82.7% → 80.3%) and the number of non-zero prototypes increasing by 9.8% (306 → 336). This suggests that the clustering attention in PACA is not merely a computational shortcut, but rather enables semantic-level feature reconstruction, allowing prototypes to cover a broader range of discriminative regions and thereby achieving higher classification accuracy with fewer prototypes. Furthermore, the accuracy curve of PIP-GN enters a pronounced plateau phase around 80.5% after epoch 60, indicating that the model struggles to escape local optima in the absence of global modeling capability.

Notably, the configuration without normalization (PIP-CLU) even slightly outperforms the full model in final accuracy (82.9% vs. 82.7%). However, this marginal advantage (0.2%) comes at the cost of training stability. As shown in Figure 4b, its accuracy curve exhibits significant “sawtooth” fluctuations during epochs 15–35, with two notable drops of 1.8–2.5%; in contrast, the full model maintains a relatively stable upward trend. More importantly, from the perspective of prototype quality, the test-set purity of the full model reaches 89.2%, significantly higher than the 86.5% of PIP-CLU (Table 2), indicating that stable feature distributions facilitate the formation of clearer semantic boundaries by cluster centers.

Based on the above analysis, the PACA module and Group Normalization constitute a complementary relationship: the former is responsible for semantic enhancement by establishing long-range dependencies through clustering attention, while the latter ensures optimization stability by suppressing feature scale drift via dual normalization. Their synergy enables PIP-PACA to achieve the optimal balance across accuracy, prototype compression rate, and semantic consistency, rather than isolated optima of individual components. This finding also provides insight for subsequent design: clustering-driven attention mechanisms exhibit high sensitivity to the stability of feature distributions, and normalization operations should not be regarded as optional auxiliary techniques, but rather as critical components for ensuring clustering quality.

### 8.5. Prototype Semantic Quality

To further analyze the interpretability of the model, we evaluate the semantic quality of the learned prototypes, with a particular focus on prototype purity. Prototype purity reflects the degree of semantic correspondence between a prototype and a specific image part, and serves as an important metric for interpretability. Beyond peak purity values, we also examine the standard deviation of purity across prototypes, as lower variance indicates more consistent and reliable interpretability across the model. We utilize the part annotations provided in the CUB dataset, specifically the ground-truth part center locations, to compute prototype purity.

**Figure 5:**
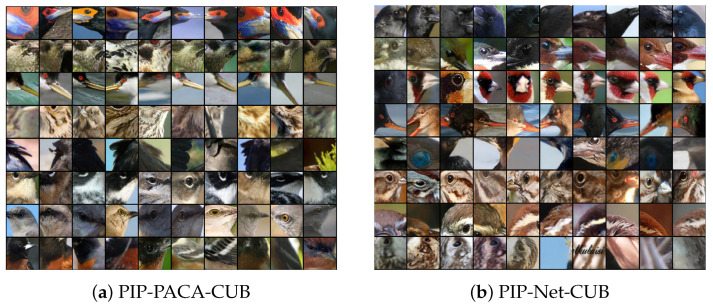
Examples of prototype examples learned by different models, with the top-10 image patches for each prototype visualized. (**a**) Visualization results of the PIP-PACA-CUB model; (**b**) Visualization results of the PIP-Net-CUB model.

For each prototype, the top-10 most similar image patches are selected, as illustrated in Figure 5. These patches are then examined to determine whether they contain the ground-truth part center. This criterion is used to quantify prototype purity. Such a measurement effectively evaluates whether a prototype consistently corresponds to a single semantic part, such as a bird’s head or wing.

As shown in Figure 5, on the CUB dataset, inconsistencies can be observed in columns 6–9 of row 8 for PIP-PACA-CUB, while more pronounced inconsistencies appear in rows 5, 7, and 8 for PIP-Net-CUB. Overall, the prototype patches produced by the proposed model exhibit modest improvements in visual consistency and alignment. These results suggest that the clustering-based attention in PACA provides modest gains in local semantic consistency. In particular, prototypes learned with PACA are less affected by background interference and exhibit more concentrated semantic representations. This can be attributed to the global clustering mechanism of the PACA module, which helps filter out local noise and strengthens responses from semantically consistent regions.

To quantitatively assess this difference, we further analyze prototype purity statistics for both models. As reported in Table 3, the average purity of the Purest (most representative region) and Overlap (most overlapping region) metrics is comparable between the two models, with differences of approximately 0.2. This suggests that both models achieve strong prototype consistency under optimal and practical conditions, indicating robust behavior.

However, the proposed model exhibits a lower standard deviation, indicating that a larger proportion of prototypes have purity values concentrated within the range of 0.7–1.0. This improvement can be attributed to the introduction of the PACA block, which enhances robustness under complex local structures. Even in scenarios where key parts are spatially adjacent or partially occluded, the learned prototypes remain highly discriminative.

Based on the above analysis of prototype semantic quality, the proposed model demonstrates a positive effect in promoting semantic understanding of local features and integrating global contextual information. It improves prototype purity while enhancing the model’s robustness to various sources of noise encountered in real-world scenarios.

### 8.6. Computational Efficiency and Practical Deployment

Section 6 analyzed the theoretical complexity of PACA, showing that clustering-based attention reduces cost from $O\left(N^{2}\right)$ to $O\left(NK\right)$. This section verifies that advantage through runtime measurements. We compare three models: PIP-PACA, the original PIP-Net, and PIP-Transformer where PACA is replaced by a lightweight Transformer block. All experiments use the CUB-200-2011 dataset with identical settings.

Table 4 reports the overall efficiency metrics. PIP-PACA adds little to PIP-Net. FLOPs rise by only 2.8% (41.40 G vs. 40.26 G), parameters by 5.0% (29.37 M vs. 27.97 M), and training time per epoch by 3.7% (69.0 s vs. 66.5 s). The full 100-epoch run takes 114.9 min versus 110.9 for PIP-Net, an increase of merely 3.6%. Inference latency is essentially unchanged at 1844 ms vs. 1862 ms. Throughput is also nearly identical (17.5 vs. 17.4 images per second). These numbers confirm that PACA’s cost is paid during training and does not burden inference.

The comparison with PIP-Transformer is revealing. PIP-Transformer demands 44.0% more FLOPs, 39.4% more parameters, and 30.8% longer training time per epoch. Its inference latency is lower (1206 ms vs. 1862 ms), but this reflects optimized matrix kernels rather than true efficiency, and it comes with 10.6% higher peak memory.

Table 5 breaks down memory usage across training. All three models show a two-stage pattern. In the first few epochs, memory stays low at around 1.8 GiB because only the backbone and prototype layers are active. At Epoch 4, the classification layer is unfrozen and the first jump occurs. PIP-PACA’s jump (+4689 MiB) is close to PIP-Net’s (+4260 MiB), but much smaller than PIP-Transformer’s (+6951 MiB). The reason is clear: PACA uses a compact assignment matrix of size N×K (with K=280 cluster centers), while the Transformer must store a full N×N attention matrix that grows quadratically with spatial resolution. At Epoch 11, all loss terms are activated together and the second jump occurs. This jump is similar across all three models, suggesting that the final stage is dominated by prototype and classification gradients rather than attention mechanisms.

The last column of Table 5 gives the Peak/Early ratio, which is the peak memory in Epoch 11+ divided by the initial memory in Epoch 1–3. This measures how much memory expands during full training. PIP-PACA achieves the lowest ratio at 9.71, meaning its final memory use is 9.71 times the initial amount. PIP-Net reaches 10.12 and PIP-Transformer reaches 11.38. Despite adding a global module, PIP-PACA is more memory-efficient than the baseline because the linear complexity of clustering offsets the overhead of its extra parameters. PIP-Transformer’s highest ratio confirms that quadratic attention is the main memory bottleneck, exactly as Section 6 predicted.

These results lead to two conclusions. First, PIP-PACA achieves substantial accuracy improvements with negligible deployment cost, making it suitable for applications that require both interpretability and efficiency. Second, the comparison with PIP-Transformer validates the design choice: replacing quadratic self-attention with linear clustering-based attention yields measurable savings in FLOPs, parameters, and memory, without sacrificing inference speed.

## 9. Conclusions

We propose PIP-PACA, an interpretable image classification model based on a Prototype-Aware Clustering Attention mechanism. By incorporating Group Normalization and the PACA module into PIP-Net, the model leverages learnable cluster centers to map features into a prototype space, enabling efficient global information modeling. This design is naturally aligned with prototype-based reasoning and reduces computational complexity from the quadratic scale of self-attention to linear complexity, while improving classification accuracy and stabilizing feature distributions.

PIP-PACA preserves the interpretability advantages of the prototype “scoring sheet” paradigm. A nonlinear mapping is introduced to amplify the contribution of key evidence, producing more focused global explanations and compact local decision bases. Experimental results on the CUB and Cars datasets demonstrate simultaneous improvements in global explanation compactness and Top-1 accuracy, along with substantial reductions in the number of prototypes (approximately 59.4% and 67.2%, respectively).

The proposed architecture maintains an intuitive reasoning process but still inherits inherent limitations. As noted, PIP-Net-based methods (including the proposed model) can only detect the presence of prototypes and cannot account for the frequency of prototype occurrences as a discriminative factor. PIP-PACA relies solely on image-level labels and does not require part-level annotations. The introduced normalization strategy accelerates convergence, while the clustering-based attention mechanism helps suppress background noise and enhances responses from semantically consistent regions. Following the principle of interpretability-driven design, the model integrates local features with global context to provide semantically meaningful explanations aligned with human perception. Furthermore, the current evaluation of interpretability relies on quantitative metrics (sparsity, purity) and qualitative visual inspection. Human-centered user studies, such as measuring human agreement with model explanations or task-completion accuracy with prototype guidance, would strengthen the claims regarding real-world interpretability.

Future work may proceed in two directions. First, non-fine-grained datasets lack stable part structures, making prototype-based reasoning ineffective. Incorporating holistic feature representations to leverage global shape and texture directly would compensate for this limitation. Second, integrating multimodal models to introduce semantic understanding channels—such as learning object quantity and social significance—may further enrich the interpretability framework and broaden its applicability beyond visual recognition.

## Figures and Tables

**Table 1:** Mean accuracy and standard deviation (3 random seeds). Global size refers to the number of non-zero prototype weights in the model. Local size denotes the average number of prototypes involved in a single prediction. Bold values indicate the best performance.

	Method	Top-1 Acc	Global Size	Local Size	Sparsity
CUB	PIP-PACA	**83.2 ± 0.2**	312	4	99.8
	PIP-Net	79.2 ± 0.3	316	4	99.7
	MCPNet	78.2 ± 0.2	N/A	N/A	N/A
	ProtoPNet	76.3	N/A	N/A	N/A
CARS	PIP-PACA	**89.1 ± 0.2**	252	2	99.8
	PIP-Net	82.8 ± 0.3	275	2	98.1
	MCPNet	81.6 ± 0.2	N/A	N/A	N/A
	ProtoPNet	81.5	N/A	N/A	N/A

**Table 2:** Ablation study on CUB-200-2011. PIP-PACA denotes the full model; PIP-Gn removes the PACA block while retaining dual Group Normalization; PIP-Clu retains PACA but removes normalization. The best result in each column is shown in bold. The arrow (↓) indicates that lower values are preferred.

Model	Top-1	Top-5	Proto	Sparsity	Purity	Stability	Memory
	**(%)**	**(%)**	↓	**(%)**	**(%)**		**(GB)**
PIP-PACA (Full)	**82.7**	**91.7**	**306**	**99.8**	**89.2**	High	18.4
PIP-Gn (w/o PACA)	80.3	88.7	336	99.7	87.8	High	17.8
PIP-Clu (w/o GN)	82.9	92.2	316	99.8	86.5	Medium	18.1

**Table 3:** Comparison of prototype purity of different models on the CUB dataset, reported as mean ± standard deviation over all relevant prototypes. Prototype purity is computed as the frequency of ground-truth part centers appearing in the top-10 image patches of each prototype. Purest and Overlap correspond to the optimal and practical scenarios, respectively, while Robustness Gap represents their difference.

Method	Purity (Purest)	Purity (Overlap)	Robustness Gap	Prototypes
PIP-PACA	85.53±17.3	85.31±17.3	0.22	311/293
PIP-Net	85.51±20.6	85.31±20.6	0.20	314/280

Note: The two values in “Prototypes” denote the number of prototypes under the Purest and Overlap settings.

**Table 4:** Comprehensive efficiency comparison on CUB-200-2011. Values are reported as mean (±std) over the respective training phases.

Model	FLOPs (G)	Params (M)	Pretrain (s)	Train (s)	Infer (ms)	Memory (GB)	Throughput	Total (min)
PIP-PACA (Ours)	41.40	29.37	42.1 ± 0.9	69.0 ± 11.0	1844 ± 146	16.7 ± 3.9	17.5 ± 1.4	114.9
PIP-Net	40.26	27.97	39.4 ± 0.7	66.5 ± 10.9	1862 ± 215	16.1 ± 3.9	17.4 ± 2.0	110.9
PIP-Transformer	57.99	39.00	65.4 ± 0.6	87.0 ± 16.1	1206 ± 87	17.8 ± 5.4	26.7 ± 1.9	87.0

**Table 5:** GPU memory footprint across training stages on CUB-200-2011. Peak allocations are reported in MiB. The two-stage expansion corresponds to unfreezing the classification layer (Epoch 4) and activating all loss terms (Epoch 11). The Peak/Early ratio equals Epoch 11+ divided by Epoch 1–3.

Model	Epoch 1–3	Epoch 4–10	Epoch 11+	1st Jump	2nd Jump	Peak/Early
PIP-PACA (Ours)	1892	6581	18,370	+4689	+11,789	9.71×
PIP-Net	1760	6020	17,822	+4260	+11,802	10.12×
PIP-Transformer	1802	8753	20,514	+6951	+11,761	11.38×

## Data Availability

Publicly available datasets were analyzed in this study. This data can be found here: CUB-200-2011 is available at http://www.vision.caltech.edu/datasets/cub_200_2011/ (accessed on 15 March 2025). Stanford Cars is available at https://github.com/jhpohovey/StanfordCars-Dataset (accessed on 15 March 2025).
